# Critique of everyday narco-capitalism

**DOI:** 10.1080/01436597.2022.2053776

**Published:** 2022-04-04

**Authors:** Maziyar Ghiabi

**Affiliations:** a College of Social Sciences and International Studies, University of Exeter, Exeter, UK; b Development Studies, School of Oriental and African Studies (SOA S), University of London, London, UK

**Keywords:** Everyday life, lifeworld, capitalism, Global South, drugs, social theory

## Abstract

Capitalism is not only an economic mode of production; it is also a form of life. This also applies to a historical type of capitalism, which is the capitalism founded on (illicit) drugs – in other words: narco-capitalism. The article discusses how capitalism alters life at the nexus of drug production, trade and consumption through a study of drug heartlands in Colombia, Afghanistan and Myanmar. What forms of life emerge under narco-capitalism? And how do people seek change and express agency in the exploitative conditions governed by narco-capital? To do so, the article proceeds through the following sections: first, it elucidates its definition of the ‘everyday’ as a conceptual and methodological scheme to understand capitalist forms of life. Then it uses material collected from people’s everyday encounter with narco-capitalism in Afghanistan, Myanmar and Colombia to discuss *mystification, predation* and *alienation*. The article explores how capitalism produces forms of life that make use of drugs and narco-capital to dispossess and alienate collectivities. Finally, the article argues that to move beyond this alienating condition, drug wars and/or development are not a solution, because drugs are not the problem. Instead, it is people’s organisation and world-building in dialectical mode to capitalist forms of life that can transform everyday life beyond predation and alienation.

## Introduction

Capitalism is not only an economic mode of production; it is also a form of life. This also applies to a historical type of capitalism, which is the capitalism founded on (illicit) drugs – in other words: narco-capitalism. What forms of life emerge in narco-capitalism? And how do people seek change and perform agency in the exploitative conditions governed by narco-capital?

The article discusses how capital alters life in the nexus of drug production, trade and consumption through a study of drug heart/borderlands of Colombia, Afghanistan and Myanmar. It is based on the study of the lifeworld of people in contexts where illicit drugs have turned into commodities. It shows how drugs are not the bearer of violence, predation, alienation; rather, these latter are part and parcel of capitalist forms of life. By form of life I refer to the experiences and cultural imagination that distinguish one’s life from other ways of being in the world. After defining what narco-capitalism is and how it connects to Capitalism, the article explains how the framework of ‘the everyday’ can help us to comprehend a capitalist form of life. The article, which is based on a large data set of primary material collected in drug borderlands, is structured around three analytical moments:

1. A definition of narco-capitalism and how we can understand it through the frame of lifeworld and forms of life.
2. A discussion of capitalist forms of life: demystification of capitalist dichotomies and truths (war/peace, chaos/government, money/drugs); the logics of predation; and alienation.
3. Conclusion reflecting on the prospect (or not) of emancipatory agency beyond narco-capitalism in drug heart/borderlands.

## What is narco-capitalism?

Since the nineteenth century, the lifeworlds of drugs have been entwined with that of capitalism. Capitalism’s rise was linked to the trade and commerce of opium in East Asia, which not only gave form to the world’s foremost empire, Britain, but also inaugurated the birth of capitalism in China (Trocki 1999, 2002). This history of narco-capitalism is one of great transformations on the world stage, made of wars, diplomacy, inter-continental and oceanic trade, and movement of capital – drugs. But how has capitalism – and narco-capitalism as a superlative expression of it – shaped the everyday existences of those in key production and trade sites in the Global South? What forms of life emerge under the capitalism of illicit drugs? By forms of life I refer to the idea that capitalism is more than an economic mode of production; as a transformative force, capitalism shapes the relationship between humans, the development of people’s potentials and their actions as interwoven with culture, emotions and desires, but it also triggers the degradation of life intrinsic to it (see Jaeggi 2018; Fassin 2018).

Forms of life in narco-capitalism rest upon different registers, ideational and material. I argue that the three main drivers in shaping human existence in this setting are *mystification, predation* and *alienation*. In the everyday lifeworld of drugs, capitalism gives way to forms of life specific to it, but not autonomous to capitalism as a whole. Drugs, after all, are commodities or entities that might easily escape the everyday. Unlike salt, sugar, milk, bread or even wine, drugs are thought of as non-essential in the recurring cycles and routines of human life. Popular and governmental treatment of illicit drugs portrays them as superfluous to everyday human activity, though drug scholarship has long demonstrated that drugs, like pharmaceuticals, are everyday technologies of health management and the line between them is blurred (Herzberg 2020; Ghiabi 2021). As quintessential everyday capitalist products, drugs are a defining commodity in producing capitalist forms of life, because they are embedded in everyday practice, from profit to punishment, and in consumption.

Historian David Courtwright refers to this human condition as ‘limbic capitalism’, in reference to the part of the brain stimulating the pursuit of pleasure through chemical inputs and undermining control of appetite (2019). Although drugs have *de facto* been part of human history since the start, accessibility to them for the willing purpose of alteration of one’s psycho-physical state has never been as easy as in contemporary capitalist times. This is what sets apart Courtwright’s limbic subgenre of capitalism. Conversely, I argue that there is no distinction between regular capitalism and its limbic manifestation, or for that matter narco-capitalism. Both engender forms of life governed by the forces of mystification, predation and alienation. The rest of this article dwells on how these forces remould human life (and, though beyond my remit, ecological life).

The association of narco-capitalism with capitalist mode of production is generally dismissed because narco-capital seems not to conform to capitalist modes of production, the governing axiom of Capitalism. For instance, most farmers cultivating coca and poppy own the land they cultivate – though often without legal title – and possess means beyond their individual labour power. They employ other peasants and migrant labourers under seasonal exploitative conditions; they negotiate, marginally, the cost of the harvest and often organise into unions. However, the production of commodities (drugs) does not earn them an actual capitalist surplus, which one would expect from those owning the means of production. Surplus, instead, is extracted through those controlling, or at least managing, the value chain where drugs pass from being plants under cultivation to high-profit goods, especially once passed across international borders into Northern markets, or into national metropolises. Managing this value chain requires the use or the threat of *violences* (Pearce 2019) to compete in the unregulated hyper-liberal market generated by the drug wars. Violences become a means of production of value affecting the everyday human relations in the lifeworld of crop cultivators, smugglers, dealers and consumers. Through different case studies, the article critiques this multi-scalar reality called narco-capitalism.^1^

However, narco-capitalism is not just an all-powerful, hegemonic structure reducing individuals and communities to impotence or passivity. Drug lifeworlds are made of the work of crop cultivators and smuggler traders, of the agents and infrastructures of repression of drug warriors, and of the experience of drug users as well as their pursuit of health and well-being. Coalescing with these, there are brokers, chemists in labs, custom agents, lawyers, vigilantes and intelligence officers recruiting workers of narco-capital at different levels of the drug lifeworld. There are also ethical entrepreneurs to seduce the public with the Pandora’s Box of drug regulation or artists and influencers of all sorts promoting nar-co-culture and the heroism of cartel leaders (Muehlmann 2013, Chapter 3). Because of this complexity, the narratives of workers, consumers and patients is essential to the understanding of everyday capitalism and its effect on drug lifeworlds. In all these activities, people have agency and pursue, through the organisation of everyday life, ways to survive, improve their status and make money without exposing themselves to excessive violence (Miller 1996; Gundur 2020). This agency, as the article highlights, can be constructive or destructive, selfish or collective, governed by contradictory pretentions, different purposes, diverging worldviews. But it rarely leads to creating circumstances that go beyond capitalist forms of life, to emancipation. As discussed in the conclusion, emancipation happens only when communities attempt world-building not as rhetoric but as practice outside of capitalist forms of life. Altogether, mystification, predation, alienation and emancipation co-exist in a tense and dialectical relation, overlapping in and co-producing the everyday. They create conditions that are oxymoronic, reproducing the ‘underlying, inescapable contradictions that animate political life and on which politics is ultimately constructed’ (Ghiabi 2019, 9).

But what is the ‘everyday’ and how can we think *with* an everyday critique?

## Everyday lifeworlds: methods, ethics, concepts

Narcocapitalism requires demystification based on a critique of the everyday in its discursive and material aspects, in the way it is thought and spoken of, and the way it affects logistics and infrastructures. As discussed by Lefebvre, ‘[e]veryday life functions within certain appearances which are not so much the products of mystifying ideologies, as contributions to the conditions needed for any mystifying ideology to operate’ (1991, 185). But where does the ‘everyday’ originate? And how does it shape the encounter of drugs lifeworlds and capitalist forms of life?

As an expression, the everyday is as generic as it can get. It is thought of as being without content or as having a content with little or no value for analysis. It is hard to come up with a final definition because different authors give heterogenous meanings to what is an everyday life approach along different conceptual lines (Gregory 1999; Highmore 2002; Steege et al. 2008). But, in principle and along the disciplinary spectrum, there are two contrasting tensions that can be identified when speaking of the everyday: one is the rhythmic repetition of events that animate life day after day; the other is the radical unpredictability of the quotidian in which inconspicuous or grand occurrences transform the flow of life. The everyday can be dull, but it can also be epic.

This contradiction lies on the surface of another more profound tension that underpins the scholars’ work on the everyday: a radical empiricism concerned with ways of being in the world and ways of transforming the world. Thus, its detractors have labelled the study of everyday life anti-theoretical, anti-intellectual and anti-Enlightenment (Templer and Ludtke 2018). I use the category of ‘everyday lifeworld’ as an organic-intellectual attempt to tackle phenomena posing theoretical problems, a solution to which may be found only empirically (Wierling 2018 [1995]), through the grassroots knowledge of those involved in its daily mechanics.

Arguably, the most influential conceptualisation of everyday life comes from Lefebvre’s Critique of Everyday Life. According to his definition – Lefebvre had Bertolt Brecht’s plebian art in mind – everyday life is defined by contradictions: ‘illusion and truth, power and helplessness, the intersection of the sector man controls and the sector he does not control’ (1991, 40). The quotidian had a spontaneous, unique dimension and a complex profound one. To understand how capitalism works, Lefebvre argued, we need to study and understand social phenomena as the result of these two sides (1991, 79). Reinstating the everyday in the study of narco-capitalism is therefore an invitation to reconsider the forces animating human agency as embedded in macro-scale phenomena. Not necessarily influential, the everyday is intimately connected to other scales and to the macro-dimension of capitalism as a global force. The territorial distinction between local/everyday, global/distant is disrupted, connecting to a ‘non-scalar’ interpretation of contemporary capitalism or to a multi-scalar one.

Here, the everyday is an attempt to study capitalism ‘from the everyday’ as produced and escalated through the work of drug producers, traders and consumers in their lifeworlds. I emphasise that narco-capitalism is not a capitalist subgenre, the gerrymandered product of discrete ‘capitalisms’ (cf Tsing 2011), but is part and parcel of the combined and uneven process of the evolution of capitalism (Bernstein 1986), often described as ‘neoliberalism’ or ‘late capitalism’. It includes fragmentation of the workforce, deterritorialization, outsourcing and the symbiotic exploitation of non-capitalist forms of production (such as that of coca and poppy growers) by predatory capitalist agents. Middlemen and mediators are the connective tissue in the economy of scale where risk is not homogeneously distributed across the drug supply chain (Bourgois 2003): middlemen, whether dealers in urban or border settings or among farmers, face fewer risks and higher economic returns compared to the end chain of street dealers, hand-picking peasants, or mules approaching and passing through international borders.

The article illuminates these junctures of everyday narco-capitalism based on primary data collected through a database of more than 60 in-depth interviews carried out in the drug producing, trading and consuming borderlands of Colombia, Afghanistan and Myanmar. Specifically, the case studies include coca grower communities *(cocaleros)* in Putumayo (Colombia); communities involved in poppy cultivation, opiate and meth consumption in Kachin and Shan states (Myanmar); and poppy growers, opiate traders and local communities in Nimroz and Nangahar (Afghanistan), between 2018 and 2020. Our interlocutors were peasants, community leaders, local and transborder traders, drug consumers, police officers, and current and former militia members. They were not necessarily or exclusively active agents of the drug economy, but rather part of the lifeworld of narco-capital. The interviews explored a broad set of themes, including life trajectories, economic and social organisation, affective and political developments, everyday practices of work and relations with local and international agents.

Access to these locations posed concrete challenges for researchers and informants; as a team, we addressed these challenges through an accurate assessment of risks, and we minimised exposure through anonymisation and mitigation strategies, in collaboration with local stakeholders in data gathering and evaluation of access strategies. Local partners, aware of contextual sensitivities and security aspects affecting in each country, carried out interviews in coordination with the project team. Research ethics was addressed by the project’s Ethics and Security panel, with representation across the partnership; synergies, in-field compromises and cross-country learning were developed through discussions across the three countries, given that research practices, customs and data protection as well as ethics requirements are deeply contextualised and may differ from UK standards. To guide these aspects, the article is informed by the data management guidance developed by the project and the security measures implemented at each site (Van Den Eynden 2021). (Names, details and acknowledgements in the article are fully anonymised to protect participants).

Moreover, the findings were put into perspective through the project’s own construction of life stories and borderlands biographies, which rely on further engagement with the field through interviews, participant observation, and visual methodologies through Global Positioning System (GPS) imagery (Goodhand, J., P. Meehan, F. Thomson, M. Ghiabi, and L. Ball 2020). Rather than mobilising abstract knowledge, the article brings to the fore the lives of those making narco-capitalism real: workers, traders, enforcers and profiters, and bystanders who suffer and benefit from the drug war. This is a methodology that opens new avenues into the study of capitalism through the everyday dispelling of spatial and temporal distinctions: of ‘centre’ versus ‘periphery’ as well as ‘history’ versus ‘forgotten (hi)stories’, ‘main-stream’ and ‘voiceless’. The everyday turns the borders, the margins, the peripheries into where the heart of social phenomena, of the state, of globality beats. Indeed, the three borderlands I refer to in the article are responsible for more than 90% of global illicit opium and heroin production and more than 50% of the world’s cocaine production’ (Bhatia et al. 2021). These spaces and these times become formative of micro- and macro-historical processes; they are not incidental to them.

Beyond Lefebvre, the everyday has also been the defining element of a vibrant historiography, centred around the late German historian Alf Lüdtke (1943–2019) and the *Alltagsgeschichte* school. Two main questions influenced their approach: the nature of everyday life under modern capitalism, and the efficacy of human agency in the face of over-whelming power (particularly during Germany’s Nazism) (Brewer 2010). To understand the lifeworld of narco-capital, I take up Lüdtke’s advice ‘not to get bogged down burrowing too deeply solely in local plots and “fields”’ (Templer and Ludtke 2018, p. 29). Instead, by reporting their own analysis of on-the-ground changes and everyday struggles, I transpose the experience and knowledge of local people in different ecologies of the Global South into an inter-regional, transcultural contexture. I am conscious that the use of voices and vignettes pinned down on the global map of capitalism may be overwhelmed by the hard data of policymakers and economists; but a critique of everyday capitalism can only occur when emerging from those turning the wheel of the machine of capital and being also its primary sacrifice (Freire 2018).

To express it with an allegory, the task of the *everyday scholar* is akin (but frankly less burdensome) to the work of the proletarian in a factory plant:

On the one hand it tends to overwhelm and crush the (individual) proletarian [scholar] under the weight of the toil [world], the institutions [metrics] and the ideas [theories] which are indeed intended to crush him. But at the same time, and in another respect, because of his incessant (everyday) contact with the real and with nature through work, the proletarian [scholar] is endowed with fundamental health and a sense of reality which other social groups [scientists] lose in so far as they become detached from practical creative activity. (Lefebvre 1991, 163)

## Mystification and demystification

To get to the everyday, the first step is to go beyond the mystified image standing in front of narco-capitalism. By mystification I refer to a reality in which that which exists is fogged by what is said to exist, by what is claimed or presumed to exist. I identify three main forms of mystification (although there are many others that can be ascribed to narco-capitalism): drug war versus drug-free peace; chaos caused by nonstate organisations versus good government produced by the state; and money producing development versus drugs preventing it.

### First mystification: war and peace

When referring to drug wars, the condition of war is often meant as a metaphor for opposition, it is a mystified reality. But those exposed to the everyday drug wars (eg users, cultivators, petty traders and their communities) know all too well that the condition of war is actual and material (Muehlmann 2013; Zigon 2018). Because these social categories are in touch with the everyday – indeed, *they* are the everyday – they can see through the fog of capital mystification, the hegemonic ideology of drug wars. Mystification is the process by which drug wars are fought for peace and order, for *development*, while their reality is that of dismembering communities and fomenting the profits of capital, in the form of drug business (narco-capital) or its laundered investment in legitimate enterprises. Furthermore, as Paley shows (2014), the drug war comes with lucrative profits for corporate interests and the state in sectors such as the petroleum and mining industries, while oiling the wheels of financial banks (Ballvé 2019). By integrating the individual, subjective experiences of people living in drug wars, it is possible to undermine the mythologies and mystification of everyday capitalism.

For instance, narco-capitalism expands during conflicts, in spaces and moments of destabilisation. But the contrary is also true. The paradigmatic case is that of Afghanistan prior and after the 2001 US-led invasion, which besides the surreal pledge to uproot the Taliban, was also carried out on the promise of eradicating drugs. Before 2001, the opium economy had increased over the decades, except in the year 2000 when the Taliban government put a controversial ban on poppy cultivation, almost zeroing Afghan opium production. Following the Anglo-American intervention in 2001 and from then up to 2021, opium production has reached previously unthinkable heights, from ca. 74 metric tons in 2000 to 7400 metric tons in 2007, and 6400 metric tons in 2019 (Bjelika 2020). The ‘peace’ brought by the Western intervention had coincided with unprecedented levels of poppy cultivation.

In Myanmar, the PA-O ceasefire agreement in 2015 coincided with the arrival of drugs and the increasingly public visibility of ‘addiction’ to substances other than opium. In Kachin state, an elderly peasant dispelled the axiom of ‘peace equals fewer drugs’ in these words: ‘Since the ceasefire period, the Chinese came in, people could move around more easily, and drugs started flooding in. Yes, the ceasefire was a good thing but since the ceasefire, drugs became more available. I see it that way’ (Interview with Elderly Peasant in Chipwi, Northeast Kachin State. Chipwi, Northeast Kachin State, 2018).

Another villager and local representative in southern Shan state, a region bordering China, Thailand and Laos, claims that there were ‘a few poppy plantations in some of the villages around Taunggyi, in Pinlaung and Hsihseng’, but following the ceasefire these territories came under the militias’ control, and ‘poppy plantations and drugs were allowed without any restrictions’. They impose taxes on the local farmers for their crops and land; ‘people would grow poppies since they can earn more money more quickly compared to other regular crops’; since then heroin and meth became widely available (Village Official 2019).

The same goes for Colombia’s cocaine and Afghanistan opiate industries; or the creation of the North American Free Trade Agreement (NAFTA) stimulating drug trade across Mexico (Muehlmann 2013). Peasants and working-class people are aware of how capital generated from drugs shapes everyday life and politics. A *cocalero* social leader in Putumayo, Colombia’s south-western region, is adamant about depicting the nexus between narco-capital and post-peace deal settlements. ‘Money depoliticises and creates Mafias’, they say, adding ‘Well, yes, because when there is disarmament [as for the Fuerzas Armadas Revolucionarias de Colombia - Ejército del Pueblo (FARC-EP)], groups called mafias enter the game’. The FARC-EP was made up of people who had been present in the territory, with local networks and knowledge/savvy; they had developed a strong connection (though fluid and controverse) to the *pueblo*, the common people. But ‘they are the same ones who today rearmed now in the service of the Mafia …. They are the same people who were FARC before and now they call themselves Mafia, a new paramilitarism [paras]’ (Community Leader 2019).^2^ In this way, former members of militant ideological groups who preached a people-oriented politics – a politics of the everyday – reconfigured their strategies in the wake of peace with the state, adopting the logics of predation. This is not to say that those among rebel groups such as the FARC-EP refusing to disarm are doing so because of the appeal of narco-capital, but that narco-capital affects modes of depoliticisation; it does not necessarily produce one single outcome (Gutiérrez and Thomson 2021).

### Second mystification: chaos and government

A general assumption is that territories under narco-capitalism are unruly and ungovernable (Rabasa et al. 2007; Ballvé 2019). But the absence of governmental rule does not coincide with a lack of authority and a void of power, nor does it exclude state connivance and co-production with narco-capital. Indeed, narco-capitalist territories suffer from an abundance of rule and an excess of authority, rather than a haemorrhage of political control. As Ballvé argues, ‘[s]uccessful drug trafficking requires particularly muscular forms of territoriality that almost instantly constitute alternative forms of political authority … woven into the very fabric of everyday life’ (2019, p. 216). So, territorial projection is as crucial as the flow of commodities along drug routes, even though it operates with differing degrees of public visibility and force in advanced capitalist states. This produces development and infrastructure, as a growing number of scholars argue (Buxton, Chinery-Hesse, and Tinasti 2020; Goodhand et al. 2021), while it also employs large swathes of the population, as in the case of Colombia and Afghanistan, in direct production and indirect services to the industry.

**Figure 1:**
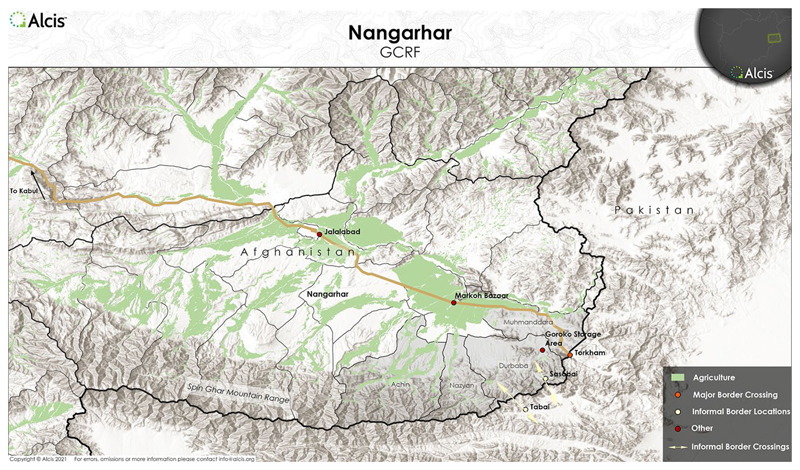
Map of Nangahar, Afghanistan. Map provided by Alcis Ltd for the Drugs & (dis)Order project, used with permission.

The drug lifeworld is not a lawless land of disorganisation; it can establish bureaucratic-type control, whereas the state itself, beyond its mystified image, may appear to be a lawless governmental machine. In Nangahar, on the Eastern Afghan border with China and Pakistan, opium production fosters governance and local economic stability in a historically insurgent territory (Figure 1). Wadaan Khan (not his real name), today a village elder in Jalalabad, after a period working abroad in 1990s, returned home where he rented a shop trading opium while the Taliban were in power. He explains: I got arrested by the Taliban as someone denounced me to them believing that I had a car, 2500 rounds of rocket propelled grenade (RPG) and a 82 mm large-calibre gun. But I had Taliban witnesses and a receipt for the weapons that I have already handed over to Taliban and those Taliban witnesses told them that I have already handed over the weapons …’. Meanwhile, he adds, ‘I continued trading opium until the end of the Taliban regime as opium was openly traded in those years’ (Interview with Pashto Tribal Elder, Nangahar – Achin District, 2018).

Later, post-2001, Wadaan Khan was asked to work in the local administration for the newly installed US-backed government of Hamid Karzai, but he rejected the offer, preferring not to be associated with either the central state or the local powerholders. After having opened a gas station and then a computer shop for his son, he became a village elder ‘solving the villagers’ problems’. Here, one can identify the competing and overlapping layers of public authority in the management of everyday life, a representation that contrasts with the unruly territories described by the top-down analysis of international observers, and one that may hold ground following the US withdrawal from Afghanistan.

### Third mystification: money and drugs

One of capitalism’s main precept is that *to have equals to be*, or to put it in terms of Karl Marx’s description of humanity in capitalism, ‘The man who has nothing is nothing’ (in Lefebvre 1991, p. 155). In capitalist regimes, to have refers to the possession of money. In narco-capitalism, drugs become money; crops of coca, opium, ephedra or the chemicals synthetised into methamphetamines turn into money. This transformation from commodity into money governs the process of ‘having’ and ‘not-having’, of being someone or leaving the land, of enjoying credit or suffering debt. Contrary to what international organisations argue (e.g. UNODC 2018, 24), drugs are not the culprit of poverty and underdevelopment. They are essential capitalistic commodities at the forefront of processes of commodification that produce cash and profit – indeed, super-profit – therefore fulfilling the promise of a capitalist form of life. They are the accelerant of capitalist processes – the steroids (or rather stimulant) of capital. The luxury capitalism of narco-traffickers is one pornographic expression of it, but narco-capital enables market growth, commodification, real estate and local infrastructural investments against the grain of the downward spiral of dispossession and displacement (Goodhand et al. 2020) while also guaranteeing ambiguous forms of social recognition (Muehlmann 2013; Gay 2015), only partly and secondarily affected by the biopolitical effect of chronic drug (ab)use.

Drugs and money are thus ontologically tied together. But this liquidity melts into solid form through the acquisition of lands, buildings, infrastructures, political representation and financial legitimacy. Rather than a vacuous and temporary asset, narco-capital – including its plant-life form – is providentially resilient and durable (as a *cocalera* put it, *coca es sin verguenza* – ‘coca is with no shame’ – meaning the plant is resilient in these ecologies).

**Figure 2:**
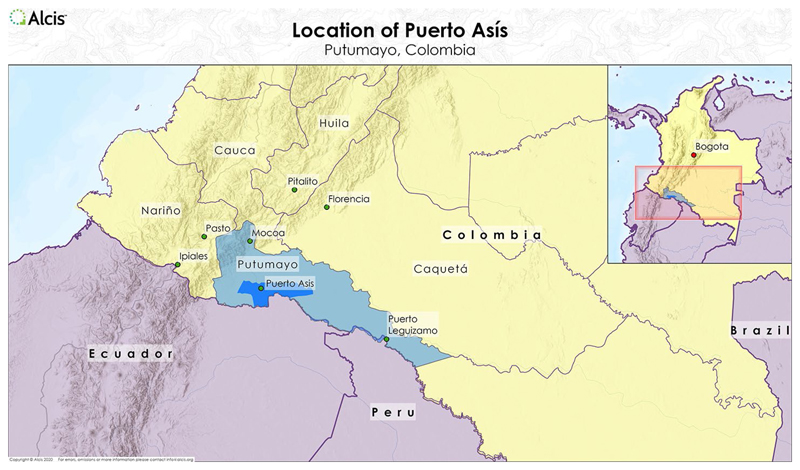
Map of Puerto Assís, Colombia. Map provided by Alcis Ltd for the Drugs & (dis)Order project, used with permission.

Indeed, coca paste trade has boosted the circulation of cash, in turn giving rise to the construction of roads and hotels, reshaping the local economy with new forms of employment. Migrants from disposed areas and landless peasants flow in search of better economic opportunities. They followed money, which in drug lifeworlds means they followed the drug (Interview with Community Leader and Former Cocalero, Putamayo, Puerto Asís, 2019). On the one hand migrants territorialise the ecology of the jungle – where there is no money to be ‘seen’, just the hand-burning toil of coca harvesting – in refuge from state-led drug war operations; on the other hand, the money generated from the plant buys things in the city, producing markets of goods and services and making sure you are someone recognised, you are (Interviews with Community leader; and Former *Cocalero* in Puerto Asís-Putumayo, 2019; Interview with Female Social Leader in Puerto Asís, Putumayo, 2019; Interview with Electrical Materials Trader. Zaranj. 2019) (Figure 2). In other words, drugs produce reterritorialisation and urbanisation in the form of capitalist development. A *cocalero* in Puerto Assís explained that ‘yes, people left [their towns] to continue cultivating coca … where it can be grown, some have already gone to Peru …. From Leguizamó down … La Laguna, Alegrías, El Canto … for all these areas, there are people from Colombia planting coca on the Peruvian side’ (Interview with Community Leader and Former Cocalero, Putamayo, Puerto Assís, 2019), deep in the jungle. Another explained that ‘here [in the city] there was a tremendous movement of money, you could see a lot of money [se *miraba mucha plata]*. When there is coca, there is work that made people come … hotels, restaurants, when there is coca there is movement’ (Interview with Former Cocalero in Putamayo, Puerto Asís 2019). Hence, the pursuit of narco-capital produces what Didier Fassin refers to as ‘forced nomads’ (2018, 41–42), not unlike the forced nomadism of low-wage workers from depressed economies towards advanced capitalist markets.

**Figure 3:**
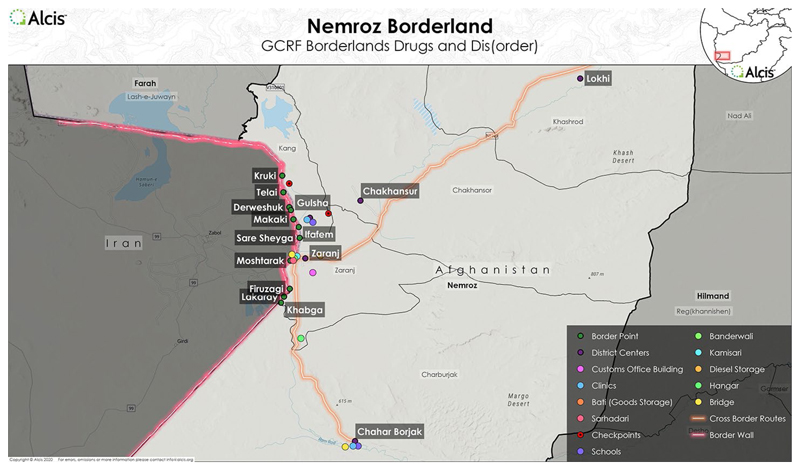
Map of Zaranj, Afghanistan. Map provided by Alcis Ltd for the Drugs & (dis)Order project, used with permission.

Along Myanmar’s borders with China, narco-capital, in tandem with the broader extractive industry of jade and other minerals, has fuelled the growth of boomtowns that become money-making machines of their own, fostering developmental processes in otherwise historically peripheral territories (Meehan 2020). In Afghanistan’s south-west region of Nirmoz, bordering Iran, towns such as Zaranj, after decades of idle non-development and stagnation, became major trade centres for the flow of commodities, including opiates, but also as part of the human trafficking route towards Europe (Mohammadi and Sadeghi 2019) – even more so following the Taliban takeover in August 2021 (Figure 3). Gol-Mohammad (not real name), a resident of Zaranj, explains that while drug traders and local elites enriched themselves, the rest of the people were affected by the combination of drought and the wall constructed on the Iranian side of the border to stop smuggling (Interview with Education Manager in Kang District. Nirmoz. 2019). The local economy pivots on the smuggling networks, with illicit drugs being a high-profit but not exclusive commodity. A decline in narco-capital, however, would correspond to the region’s shrinking livelihood and limited monetisation.

## The logics of predation

Disruptive and productive, narco-capital shapes the lives of people living and working in the cycles of capital accumulation and annihilation. Indeed, capitalist lifeworlds are necessarily predatory, while drug lifeworlds can escape the logics of predation, inasmuch they defy capitalist forms of life.

Marx identified the origins of capitalism in the primitive accumulation of capital – in the form of land, for instance (2013). This almost mythological origin – set in a moment like Eve eating the apple – determined the dialectics between those who own capital and those who sell their labour to survive. This imbalance has not put an end to the limitless need to profit or to increment accumulation. It has taken form in what Bourgois refers to as ‘predatory accumulation’. According to him, predatory accumulation relies on the ‘unusable labour of increasingly lumpenised populations expelled from the licit economies of the Global North and South’ (2018, p. 390). The reserve army of un(der)employed and precarious workers and peasants sustains capitalism’s incessant need for profit, but in practice predation turns into a form of life where people seeking better livelihood become a disposable force for the toil of narco-capital, meting out or being met with violence.

Predation affects everyday life in multifarious ways. Peasants cultivate cash crops such as poppy or coca because of the necessity to generate cash (rather than agricultural goods for the benefit of local communities (Interview with Female Social Leader in Puerto Asís, Putumayo 2019). But they are consequently dispossessed of their lands either because of drug war operations, such as aerial fumigations or actual armed eradication (Rhodes et al. 2020; Interview with Electrical Materials Trader. Zaranj 2019), or due to the unrestrained violence of landowners’ militias, state and imperial armies (mostly US) and drug cartels. Equally, traders and petty dealers face the militarised onslaught of governments and rival organisations in the drug wars and/or the war on insurgency. People deprived of social bonds and community become the target of the greed of the pharmaceutical industry or of predatory dealing practices in its promotion of opioids and other habit-forming substances (Bourgois 2018; Keane 2011). In Myanmar, for instance, toilers of the extractive industry use large quantities of heroin and *yaa baa* (meth) to cope with working conditions. Human agency couples with the more-than-human agency in the chemistry of meth to generate the physical and psychological conditions to sustain otherwise inhumane circumstances of life.

Again, it is not the drug *per se* that is a source of alienation or dispossession, as it is reiterated in drug war parlance or the hegemonic everyday anti-drug rhetoric. Rather, it is the capitalist forms of life that undermine life through predation. In Danai in Western Kachin State, a miner explained how drug use in the amber mines is the rule for workers. The nature of the work is extremely tiresome so that when he inhales heroin or *yaa baa*, he becomes ‘so energetic’. He confesses that, over time, he witnessed many cohorts ‘becoming addicts’ or ‘perishing because of drugs’. Opium, a softer narcotic that would have fewer adverse habit-forming effects, is ‘hard to get’ but heroin and meth are ‘found easily’ as they perform more effectively in these labour conditions. But the intimate link between predatory work setting and drug use habit is explained in his closing sentence: ‘Many miners ruined their lives *due to the nature of the mining work’* (Interview with Miner in Kachin State. Miner, Danai Township, Kachin State 2018) – not the drugs.

It is the nature of work, of predatory exploitation, that brings ruin to miners’ lives. The extraction of value through exploitative labour makes tactical the distribution of stimulant drugs meant to increase productivity and therefore capital accumulation. The overlapping of the predation of labour extraction with the predation of ‘addiction’ is a condition that the miners understand well. One of the miners confirmed that the ‘owner of the mining business gave *yaa baa* pills to the stone workers’, the heavier toll in the mine. The drug, the owner claimed, ‘make[s] them stronger and healthier’. ‘Sometimes they cannot even hire workers if they cannot provide them with drugs’, claims a miner, who adds that once ‘addicted, the workers buy the drugs with all the money they make each day …’ (Interview with Miner in Loilem Township, Shan State 2018). Surplus is gained through the exploitation of labour, but one can imagine that it is also the outcome of debt incurred because of chronic drug use.

These vignettes are not singular to Myanmar’s mining industry. There is a consonant ethics in cocaine and opiates (and meth) becoming more widespread among the working class and younger generations (including workers in the drug economy), in Colombia and Afghanistan, respectively (as elsewhere), where drug consumption was previously marginal. Parallel phenomena occur across the Global South and North, for instance in workers doing long night shifts drinking unwholesome quantities of energy drinks; students and academics taking performance pills; truck drivers using stimulants for their long journeys; traders snorting cocaine to seek alertness; and other forms (Ghiabi 2019; Pine 2007).

Predation is a quintessential capitalist form of life. Predatory accumulation affects workers producing narco-capital, but it equally shapes the business of oil, land, precious minerals, human trafficking and extortion or taxation. That is why land-owning elites, prosperous traders, criminal organisations and governments cooperate actively (or condescend passively) with the removal of grassroots social groups and community organisers who perform world-building activities. Indeed, the most dangerous role in narco-capitalist lands is that of grassroots activism or social leader *(lider social* in Colombia), because, as one of the latter puts it, ‘it is not convenient for the government [or the narcos] to have an organised people demanding its rights’ (Interview with Community Leader and Former Cocalero in Putamayo, Puerto Assís 2019). Since the 2016 peace agreement between the Colombian government and the FARC-EP, more than 800 community leaders have been killed, with increasing numbers since the outbreak of the Covid-19 pandemic (Vargas 2020). This is testimony to the predatory logics of everyday capitalism, in its encroachment in pursuit of narco-capital but also with respect to profit and assertion of control beyond the realm of drug business, as evidenced during Colombia’s anti-government protest of 2021. More than anything else, the logic of predation works against everyday world-building alternatives to narco-capitalism, or to the oil industry, the jade trade, environmental destruction or human trafficking. Those, such as the Colombian social leaders, who oppose the existing organisation of narco-capital – and hence of capitalism in their territories – face the predation of the state, the mafias, the paramilitaries and/or the drug cartels. The apparent antagonism between these violent forces is the ideological mask covering their collusion in enforcing predatory accumulation.

## Alienation as a form of life

The logic of predation governs narco-capitalism from below. Its prime mover and rationale is money, ‘the human being’s *alienated* essence’ (Lefebvre 1991, 181, italics added). Alienation is a concept central to the understanding of everyday narco-capitalism because it connects the question of commodity to that of consumption and healing (from ‘addiction’). In the drug borderlands I consider, the peasants own the land, often without formal titles, where they cultivate the poppy or coca. But the monetisation of the crop only occurs through the mediation of predatory actors, the larger landowners or the smuggling cartels acting as a capitalist class that can extract value by moving commodities across contested spaces, including borderlands or frontiers. The crop is often purchased before harvest; in fact, it ‘belongs’ to those capable of trading it. This is alienation’s easiest aspect to understand: the alienation of the worker from the product of labour and from the real value of work. As a commodity, drugs govern human relations and the social order; they become things that have a power on their own: a fetish. In conceptual terms, they are a reified reality independent of humans and their lifeworld (cf Lefebvre 1991, 186; Marx 2013).

In Afghanistan, foreign powers and domestic militias uprooted historical patterns of agricultural production, leaving only small avenues other than poppy farming as a source of capital (Mansfield 2016). Concomitantly, foreign invaders have waged war on poppy cultivators and meth lab workers, through aerial bombardment or by outsourcing the fight to national anti-narcotic institutions. Militant groups, such as the Taliban now in government, have extracted taxes from the economy to sustain the insurgency against the state, which eventually succeeded. To the alienation from economic life corresponds an alienation from the socio-political order where local communities are at the mercy of predatory forces.

However, alienation is not confined to the sphere of production and narco-capital. It pervades everyday life. Commodification and shifting patterns of consumption exemplify some of the processes of alienation. Drugs, such as opiates, cocaine and methamphetamines, are ideal consumer goods because of their habit-forming potential. Regular use of drugs is a side effect of the massive disruption caused by war, including drug wars, but also part and parcel of the biochemistry of these substances, which prosper under capitalistic forms of life. Understood in its ambiguous (though often abused) meaning, ‘addiction’ is not simply a biological or medical condition, but rather a social, human phenomenon that interlinks the intimate, biographical conditions of the individual and their community with their historical and political lifeworld. Alienation from everyday life is also the result of what is called ‘addiction’, especially in conditions where predatory forces make impossible or impede the existence of community among those who use drugs. Even from an etymological viewpoint, ‘alienation’ and ‘addiction’ bear a distant resemblance: the first indicates ‘to give up or transfer the ownership or property of something to someone else’; the second indicates ‘to enslave’ or ‘to abandon one’s freedom’. Narco-capitalism prospers in the dual exercise of ‘alienation’ and ‘addiction’, the latter becoming bio-capital, the capital generated by exploiting qualified life *(bios)*.

The drug war cohabitates with capitalist predation: internal markets in drug-producing countries such as Afghanistan and Myanmar have brought to light the nexus between producing drugs – the reified alienation of workers in the crop fields or the labs – and consumption of drugs – the social alienation of people expelled from the civic order *because* of the mystified drug war. For example, Kabul is today one of the world’s capitals with the highest incidence of street ‘addiction’; according to the Afghan National Drug Use Survey, one in ten people live with ‘addiction’ (UNODC 2021). An increasing number of women and children are using drugs, a fact that is discontinuous with the public perception of these categories in Afghan culture (NPR 2019). Afghan drug producers and traders have established a complex network of meth labs that could provide employment to up to 20,000 people (EMCDDA 2020). Yet as the industry grows and produces capital, it also introduces meth to the local population, so far mostly unaware of the drug. It displaces the traditional use of opium, with its social and cultural place, in favour of meth which has had no place in Afghan consumption culture. Returning from Iran during the 2010s, where meth became popular in the 2000s, Afghan migrants introduced consumer habits exogenous to their everyday lifeworlds. This transformation in consumption is the product of the transformative power of narco-capitalism.

Similar conditions exist across the Global South where the tandem of predatory accumulation and drug war have disrupted the lives of millions. Thiha (not his real name), a young man from Taunggyi in Myanmar, explained how alienation works in the everyday: when Thiha was 20 he started using opium which he drank mixed with cough syrup, a cocktail known as ‘Formula’. Alcohol failed him and a friend introduced him to the mix. His parents brought a doctor to see *what was wrong with him*. Thiha recalls that ‘the doctor informed them that *I had become a "junkie"*. I tried to quit by locking and tying myself up in the room’. Yet every time he tried to quit, he returned to Formula. Even now as he works in indoor electrical wiring, he regularly uses small quantities of the drink. He is aware that his bio-social status alienates him: ‘my employer, looking at my complexion, knows [that I am an addict] and pays me less than normal. I don’t like it but I can’t complain’ (Interview with Resident of Taunggyi, Southern Shan State. Taunggyi, Southern Shan State. 2018).

It is not the drug as a reified commodity that alienates, but rather the diagnosis of ‘addiction’ that confines the individual to an alienated status within the social order, where he/she is isolated, dispossessed and exploited more than other non-using workers. Alienation is experience and status, both real and imaginary, material and psychological; it is symbiotic to the capitalist forms of life. It also guarantees higher profits for exploitative employers which, on pay, run a race to the bottom. It is not unusual, therefore, that people targeted by the drug war end up being employed for the profit of capital, including narco-capital, either as precarious low-wage earners or petty dealers and ‘mules’ (Ghiabi 2020, Muehlmann 2013). After all, the drug business is, in Bourgois’s words, ‘an equal opportunity employer’ (1989, 8) even when it turns the low-hanging fruits into disposable ones.

## Conclusions: emancipation in everyday drug lifeworlds

Capitalism is predatory and alienating and, in its nexus with drugs, it rests upon a mystified reality where profit – whether to survive, to pay debts, to find a respected place in society, or to prosper with capital accumulation – governs its logics. Violence, the threat of it and the fear of it, regulates conflict between narco-capitalists themselves, and between them and the state, but it weighs upon ordinary people. This condition of permanent potential violence permeates the everyday and inhibits human agency.

The opposite of alienation is emancipation, to free oneself from the *potestas*, p. 8, ie coercive power/capital. This is a knowledge that stands clear in the mindset of everyday people. Caught between drug war capitalism and narco-capitalism, everyday people’s condition may appear helpless. By building on everyday experiences, the article offered a global comparative critique of capitalist forms of life in their encounters with drug economies. This is an incursion into unstated evidence: in the nexus of capitalism with illicit drugs, the problem is not represented by the existence (in the form of supply or demand) of drugs; capitalism and the forms of life it engenders are the problem. Existential resistance and survival do not offer meaningful alternatives to capitalist exploitation. Only organisation in dialectical opposition to capitalist forms of life can give way to world-building alternatives for those caught in the crossfire of state- and narco-capital repression. This knowledge, in itself a form of pedagogy of the oppressed (Freire 2018), gives people agency in ways that top-down research on drugs often fails to see. Incidentally, it also provides those in policy-worlds with ways to rethink the question of drugs from close up, though I am pessimistic about policy-makers’ interest in solutions for the everyday.

Often invisible to the way we portray global capitalism and transnational narco-capital, the everyday remains a creative force with the potential to generate novel lifeworlds beyond capitalism. In Colombia, *cocalero* organisations are rethinking their economic lifeworld and attempting to materialise a ‘solidarity economy’. One of the many Colombian social leaders interviewed expressed how the community clings to the territory not only because of its economic resources, but also because of ’ biodiversity, … the source of life that we have here in the Amazon’. It is the practice of world-building through ‘the solidarity economy and the issue of cooperativism’ that re-imagines the borderland’s lifeworld beyond capitalist forms of life. The social leader is not afraid to reflect and ask the thorny question: ‘why do we say solidarity economy?’ They respond by saying it is only through an alternative socio-economic order that one can think ‘about the needs of all, not a selfish economy [*una economia egoísta*]’. Because

[the plan is] to make a diagnosis through the projects and divide the production, then, if those from there produce corn and those from here produce sugarcane, for example, then we are going to manage that economy among the farmers themselves through a solidarity economy process. And we are not going to be subject to the production of the great businessman, the landowner ….We are thinking about ourselves …. (Interview with Female Social Leader in Puerto Asís, Putumayo 2019)

These communities are thinking about the everyday. And if the everyday requires the cultivation of coca or opium, then the solidarity economy will be organised around that necessity, which is a necessity of the local and of the everyday, not of the logic of predation in seeking super-profits (see ‘Community Economies’ in Kothari et al. 2019). Solidarity and social organisation are the response to capitalist predation and alienation as a form of life.

This response is tied to the experience of the everyday and to the critique of (narco)capitalism. Its expression is not singular and exportable as commodity ideas or business models. It is territorial and temporally situated, alert to material struggles and to practical challenges. It is the result of collective thinking and of everyday living against predatory forces, acting in tandem: discursively, globally, territorially, locally. It moves beyond the humanitarian compassion of foreign states and international agencies and demystifies the promises of the state.

World-building is taking place in Colombia more explicitly than in Afghanistan or Myanmar. Why is this so? Emancipatory practices learn from a critique of everyday capitalism, something that is taking place more powerfully in Colombia because local communities have stepped up social organisation and territorial reclaiming as a first step towards a different everyday lifeworld, rather than as mitigation, survival and resilience. Land redistribution, access to resources and public health, biodiversity, community participation and antagonism to the predatory state and its mystified rhizomes – such as narcos, paras, mafias and armies as well as bureaucrats and vigilantes – have occupied a central role in people’s politics. Conversely, in Myanmar, popular organisations have recurred to the moral call against drugs as in the case of militia-led anti-drug campaigns, which, although presenting itself as an emancipatory politics for rebuilding local communities, fetishises drugs as the totem of all communal and societal evils (Dan et al. 2021). By identifying drugs as the main enemy, social change fails to undermine the structure of predation and alienation affecting life across Myanmar’s exploited communities. As for Afghanistan, the direct presence of imperialist armies with an uncompromising drug war ideology has so far impeded processes of internal confrontation between the dependent state in Kabul, quickly deterritorialising; the territorial(ising) insurgency of the Taliban now controlling the state; and the multifarious communities that negotiate their livelihoods through the drug economy and the informal smuggling networks. Before the US invasion of Afghanistan, the Taliban government did not impede local communities from cultivating the poppy or producing opium (nor did it force them to). Levels of drug abuse were minimal and no spillover was reported among the general population. Opiates were dealt with as an export commodity with only marginal consumption within Afghan communities. Following the US invasion of Afghanistan, no space is left for a critique of everyday capitalism, but only expedience for survival between the drug war, the war on terror and internal colonial encroachment by powerful elites and their social networks. With the US withdrawal from Afghanistan causing economic isolation and the need for fast and easy capital, the Afghan drug economy is there to stay. Looking at the problem as being embodied by drugs or, for that matter, their nexus with religious, state, and foreign extremisms, impedes the unleashing of world-building potentials in emplacing drugs in a novel everyday lifeworld. Social and top-down interventions fail systematically. Instead, these interventions produce everyday harms in the guise of ecological damage, livelihood dispossession and chronic health conditions.

The vignettes reported here stand in a global and yet intimate relation with other cases. For instance, they connect with the activism of drug-using communities in Vancouver, who reclaim a politics of showing and the foundation of ’a community of those without community’ (Zigon 2018, p. 79); or with the lifeworld of khat traders and consumers who exist beyond the drug war and capitalist forms of life (Carrier and Klantschnig 2018). Across the Global South, in places where the stakes of confronting narco-capitalism are higher and the predatory nature of capital more virulent, there are attempts to reconfigure the everyday life-worlds of drugs. The Bolivian cocalero union leader turned president Evo Morales embodied everyday knowledge for all the people in the coca lifeworld (see Grisaffi 2018), aptly recognising the centrality of Empire in the work of narco-capitalism. History also provides some instances of world-building: between 1969 and 1979, Iran implemented an opium maintenance monopoly, *de facto* legalising opium consumption (Ghiabi 2021), not unlike the cannabis monopoly envisioned under Uruguay’s socialist president Pepe Mujica. These were in line with the existing cultural norms but also with models that ran against the predatory logic of capitalism, in its embrace of either drug war or drug commercialisation.

For people’s everyday agency to overcome the *violences* of (narco)capitalism, they need to give life to a new order of things (see Pearce 2019, 11), which means adopting a transformative approach to the political economy of land–drugs–livelihood, *to capitalism*.

## Notes

1 For a different, thoughtful definition of *narcocapitalism*, see Pine (2007). See also Gootenberg (2002).

2 The interviewee refers to ex-FARC members from the 48^th^ front joining a group called *La Mafia*, which emerged from post-demobilisation paramilitary groups, but is now apparently just an armed drug business.
